# Single-Molecule Localization Microscopy allows for the analysis of cancer metastasis-specific miRNA distribution on the nanoscale

**DOI:** 10.18632/oncotarget.6297

**Published:** 2015-11-05

**Authors:** Olga Oleksiuk, Mohammed Abba, Kerem Can Tezcan, Wladimir Schaufler, Felix Bestvater, Nitin Patil, Udo Birk, Mathias Hafner, Peter Altevogt, Christoph Cremer, Heike Allgayer

**Affiliations:** ^1^ Department of Experimental Surgery, Medical Faculty Mannheim, University of Heidelberg, Heidelberg, Germany; ^2^ Centre for Biomedicine and Medical Technology Mannheim, University of Heidelberg, Heidelberg, Germany; ^3^ Light Microscopy Facility, German Cancer Research Centre (DKFZ), Heidelberg, Germany; ^4^ Karlsruhe Institute of Technology, Karlsruhe University, Germany; ^5^ Institute of Molecular Biology (IMB), Mainz, Germany; ^6^ Institute for Molecular and Cellular Biology, Mannheim University of Applied Sciences, Mannheim, Germany; ^7^ Skin Cancer Unit, German Cancer Research Center (DKFZ), Heidelberg and Dept. of Dermatology, Venereology and Allergology, UMM, University of Heidelberg, Heidelberg, Germany; ^8^ Institute of Pharmacy and Molecular Biotechnology (IPMB), Heidelberg, Germany

**Keywords:** microRNAs, miR-31, super-resolution, localization microscopy, metastasis

## Abstract

We describe a novel approach for the detection of small non-coding RNAs in single cells by Single-Molecule Localization Microscopy (SMLM). We used a modified SMLM–setup and applied this instrument in a first proof-of-principle concept to human cancer cell lines. Our method is able to visualize single microRNA (miR)-molecules in fixed cells with a localization accuracy of 10–15 nm, and is able to quantify and analyse clustering and localization in particular subcellular sites, including exosomes. We compared the metastasis-site derived (SW620) and primary site derived (SW480) human colorectal cancer (CRC) cell lines, and (as a proof of principle) evaluated the metastasis relevant miR-31 as a first example. We observed that the subcellular distribution of miR-31 molecules in both cell lines was very heterogeneous with the largest subpopulation of optically acquired weakly metastatic cells characterized by a low number of miR-31 molecules, as opposed to a significantly higher number in the majority of the highly metastatic cells.

Furthermore, the highly metastatic cells had significantly more miR-31-molecules in the extracellular space, which were visualized to co-localize with exosomes in significantly higher numbers. From this study, we conclude that miRs are not only aberrantly expressed and regulated, but also differentially compartmentalized in cells with different metastatic potential. Taken together, this novel approach, by providing single molecule images of miRNAs *in cellulo* can be used as a powerful supplementary tool in the analysis of miRNA function and behaviour and has far reaching potential in defining metastasis-critical subpopulations within a given heterogeneous cancer cell population.

## INTRODUCTION

The majority of cancer related deaths are invariably linked to metastasis, and not to the primary tumor [1]. Metastasis in itself is a complex process and convincing evidence indicates that the tumor microenvironment is both critical and essential to the progression and dissemination of tumor cells. Several molecules and signalling cascades have been implicated in metastasis, and the pertinent question to date has been to identify not only the most crucial ones, but especially also to decipher how they interact with each other, and in different cell types to foster metastatic progression.

MicroRNAs (miRs) have recently been unraveled as mediators of a novel principle of post-transcriptional gene regulation. MicroRNA deregulation endows cancer cells with the ability to metastasize, with several miRs shown to cause tumor progression in virtually all cancer types. Some miRs, such as miR-31, are especially interesting as they are perceived to be potential master regulators of metastasis, especially in solid tumors like breast cancer [2]. Nonetheless, several conflicting reports as to their function in different cancer or cell types are abound in literature [3–5], thus asking for a more detailed analysis of their action at the single cell and single molecule level.

Recent investigations [6–8] suggest that a mechanistic understanding of the functionality of miRs during metastasis, and related molecular processes requires a detailed study of their release as well as localization patterns. More so, the subcellular association of miRs with mRNAs and their localization is seen as increasingly crucial especially in in the context of metastasis [9–11]. This becomes more relevant since miRs secreted by cells are transported systemically, and are able to prime the metastatic niche at distant sites [7, 12]. Thus, we hypothesized that miRs are not only differentially regulated, but also differentially compartmentalized in cells capable of metastasis. The validation of this principle requires advanced microscopy methods capable of evaluating the subcellular compartmentalization of miRs. However, up until now, the detection of small RNAs in biological systems with microscopy methods has been somewhat limited, in part due to poor target accessibility of designed probes, low signal-to-background ratio and the theoretical limit of resolution in light microscopy. The existing strategy of visualizing small RNAs and mRNAs with multiple fluorophore labelling, and subsequent signal amplification allows only for an estimation of relative differences [13]. Moreover, widely used methods like *in situ* hybridization are limited by diffraction [14, 15] and newer techniques using nanoparticles or molecular beacons to track these molecules in living cells also have many shortcomings [16].

We developed a novel approach to visualize and quantify single miRs, using Single-Molecule Localization Microscopy (SMLM). With this system, the use of a secondary wavelength for switching or activation of fluorophores (as in PALM or STORM) is not necessary, however, a suitable embedding medium is needed to improve blinking behaviour [17–19]. Furthermore, in our case, the SMLM optical setup was upgraded with a high-precision optical alignment (Shack Hartmann sensor) and, novel dynamics to improve the thermal and mechanical stability of the entire system. Here, we report the first single-molecule super-resolution localization microscopy approach that is able to detect single microRNA molecules with a localization accuracy of 10–15 nm, using the metastasis relevant hsa-miR-31 as a first prototype molecule. We also present our analysis of the subcellular distribution of detected miR-31-molecules, their clustering patterns and the co-localization of secreted molecules with exosomes, and for the first time show significant differences in the distribution of miR-31 molecules in human cancer cells with high and low metastatic potential.

## RESULTS

### Localization microscopy as the approach to detect microRNAs

**Figure 1:**
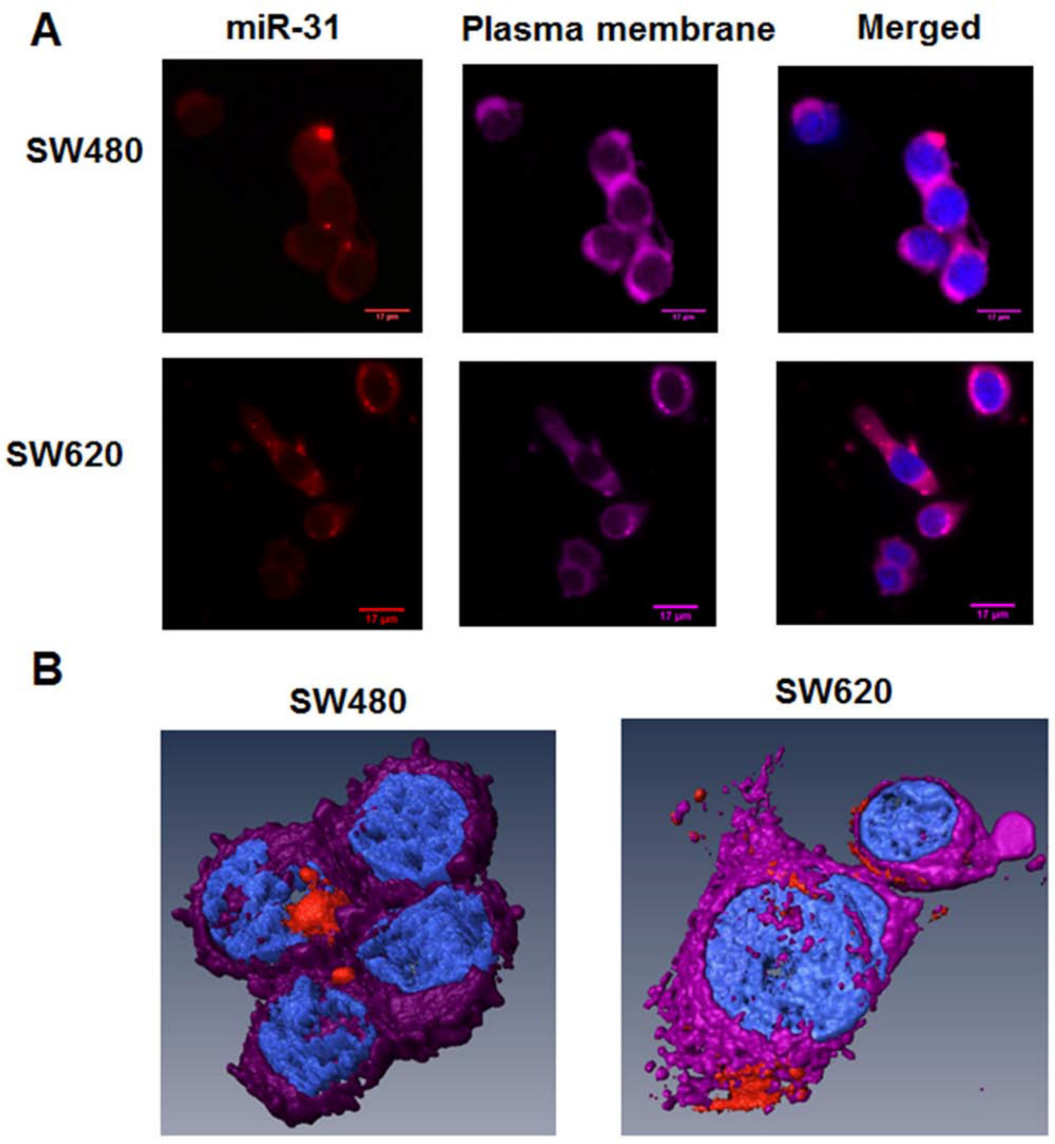
Distribution of miR-31 molecules in SW480 and SW620 CRC cells by conventional microscopy, including 3D-reconstruction of confocal images **A.** Conventional microscopy images of SW480 and SW620 cells. The human CRC SW480 (low metastatic potential) and SW620 (highly metastatic) cell lines were transfected with 10 nM of miR-31 probe-Alexa568 (red color) for 24 h. Then, the plasma membranes of cells were stained with Cell Mask Deep Red (purple color). Cells were fixed by 4% PFA and nuclei were stained with DAPI (blue color). **B.** 3D reconstruction of selected cells from (A) above.

To visualize and detect the selected proof-of-principle miR of interest, we transfected SW480 and SW620 cells with a linear RNA oligonucleotide probe, whose sequence was complementary to that of the human mature miR-31. SW480 cells are primary tumor derived cultured colon cancer cells with low metastatic potential, originating from the same genetic background as the highly metastatic SW620 cell line which is derived from a lymph node metastatic lesion [20, 21]. The probe was labelled at the 5′-end with an SMLM suitable photo-switchable Alexa568 fluorophore (IBA GmbH, Göttingen, Germany). We acquired images with conventional, including time-lapse and confocal, microscopy and observed that the probe was successfully taken up in both SW480 and SW620 cell lines with a high fluorescent signal intensity (Alexa568 probe) over ten orders of magnitude compared to both the global and local background signals (Figures 1A and 1B).

**Figure 2:**
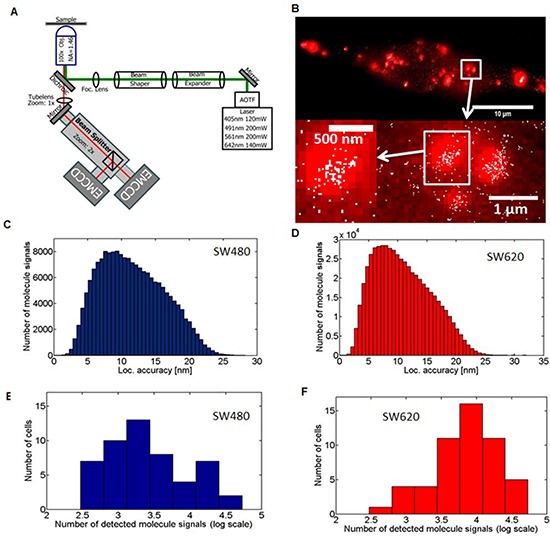
Single-Molecule Localization Microscopy and detection of miR-31 molecules in cancer cell lines **A.** Schematic representation of the SMLM optical setup used. Details of the application are contained in the materials and methods section **B.** Comparison of wide-field fluorescence microscopy (red) and SMLM (white) acquired images demonstrating markedly significant differences in resolution. The Abbe limit of optical resolution for wide-field is about 200 nm compared to effective resolution of SMLM from the localization accuracy of 12 nm. The SW480 low-metastatic and the SW620 highly metastatic human CRC cell lines were transfected with 10 nM of an Alexa568 labelled miR-31 probe for 24 h, and fixed with 4% PFA. 3000 frames of SMLM acquired images were reconstructed in home-written Matlab software. Localization accuracy histograms of SMLM acquired single cells in **C.** SW480 (*n* = 51, mean = 11.60 nm, median = 11.12 nm) and **D.** SW620 (*n* = 52, mean = 10.82 nm, median = 10.21 nm) cells. The cumulative data from 5 independent experiments are shown. **E.** and **F.** Absolute tally (numbers) of single miR-31 molecule signals detected in SW480 and SW620 cell lines. The x-axis shows the number of detected molecules/cell (log) with the *y*-axis indicating in how many cells this tally was acquired. A total of *n* = 51, and *n* = 52 cells were evaluated for SW480 and SW620 cells, respectively.

In order to acquire images, including positions of the individual miRs in fixed cells, photo-switchable visualization of the labelled miR-31 molecules was implemented. Images were acquired with a custom-built localization microscopy apparatus (Figure 2A). To achieve the intended high light intensity in the focal plane of the SMLM microscope, we used a special beam shaping system allowing for an efficient homogeneous illumination. The microscope was built using the original iMIC microscopy core (FEI Munich GmbH, Germany) with improvement of thermal stability by adding a water-based temperature control system.

We compared images acquired by conventional and SMLM microcopy with significant differences in the resolution of the images (Figure 2B). Using conventional microscopy, it was impossible to accurately obtain detailed information about the spatial distribution of the miR molecules, as it was not possible to discriminate single molecule signals inside clusters. This was, however, achievable with SMLM in both SW480 and SW620 cell lines, seen as sets of smaller structures with varying distances between single molecule signals ‘dots’, with a localization accuracy of 10–15 nm (Figures 2C and 2D, respectively).

We also observed significant heterogeneity in the number of detected single miR-31 molecule signals. The low-metastasizing SW480 cells were characterized by a few cells in the high range of detected single molecule signals (1.5 10^4^–2.5 10^4^ molecule signals/cell) and several cells in the low range of 10^3^–1 × 10^4^ molecule signals/cell. In contrast, the highly metastatic SW620 cells showed a much higher total number of single miR-31 molecule signals than the SW480 cells, with a significant number in the very high range of 2.5 × 10^4^– 4 × 10^4^ molecules/cell (Figures 2E and 2F). This variation in the number of detected molecule signals can be explained by the heterogeneous expression of miR-31, as would be expected of the heterogeneous diversity of individual cancer cells. Still, the highly metastatic cancer cells on average showed a significantly higher number of miR-31 molecules as compared to their low metastatic counterpart. The transfection efficiency was not significantly different between these cell lines (Supplementary Figure S1).

To validate our results and confirm the specificity of detection, we proceeded to do a series of experiments. First, by using RT-PCR, we quantified the levels of miR-31 in transfected and untransfected cells. For relative quantification, Ct values were normalised to either RNU6B or small nucleolar RNA SNORD72, and expression levels were equally compared across diverse cell lines including those with minimal endogenous expression [22]. The different cell lines were characterized by varying levels of miR-31 endogenous expression (Supplementary Figure S2A).

Additionally, as further proof of probe specificity, we conducted luciferase reporter assays in SW480 and SW620 cells using the 3′UTR of cMET mRNA, which is a validated target of miR-31 [22]. Ideally, our miR-31-Alexa568 probe binds to endogenous miR-31 molecules in the cell, and consequently, less miR-31 is available to repress the reporter luciferase gene, meaning that in comparison to a scrambled oligonucleotide control, the reporter activity is increased. This enhanced luciferase activity together with our RT-PCR experiments, confirms that our probe with Alexa568 is specific to miR-31, and at least at the c-Met mRNA, exerts a similar function as the endogenous miR-31 molecule (Supplementary Figure 2B).

Finally, to confirm if the principle and specificity for detection was applicable to other miRNAs, we designed a probe complementary to another metastasis-relevant miRNA, miR-21, also tagged with Alexa568, and measured the same parameters that we evaluated for miR-31 in SW480 and SW620 cells, and an additional control cell line (WiDr). The number of detected single molecule signals were comparable to the relative expression levels (as measured by RT-PCR) in all 3 cell lines (Supplementary Figures S3A and S3B). Next, we evaluated the localization accuracies of miR-21 in all 3 cell lines, which were also similar to those of miR-31 (Supplementary Figure S3C).

### Cluster and segmentation analysis of subcellular localization of miR-31 in metastatic versus non-metastatic human colorectal cancer cells

**Figure 3:**
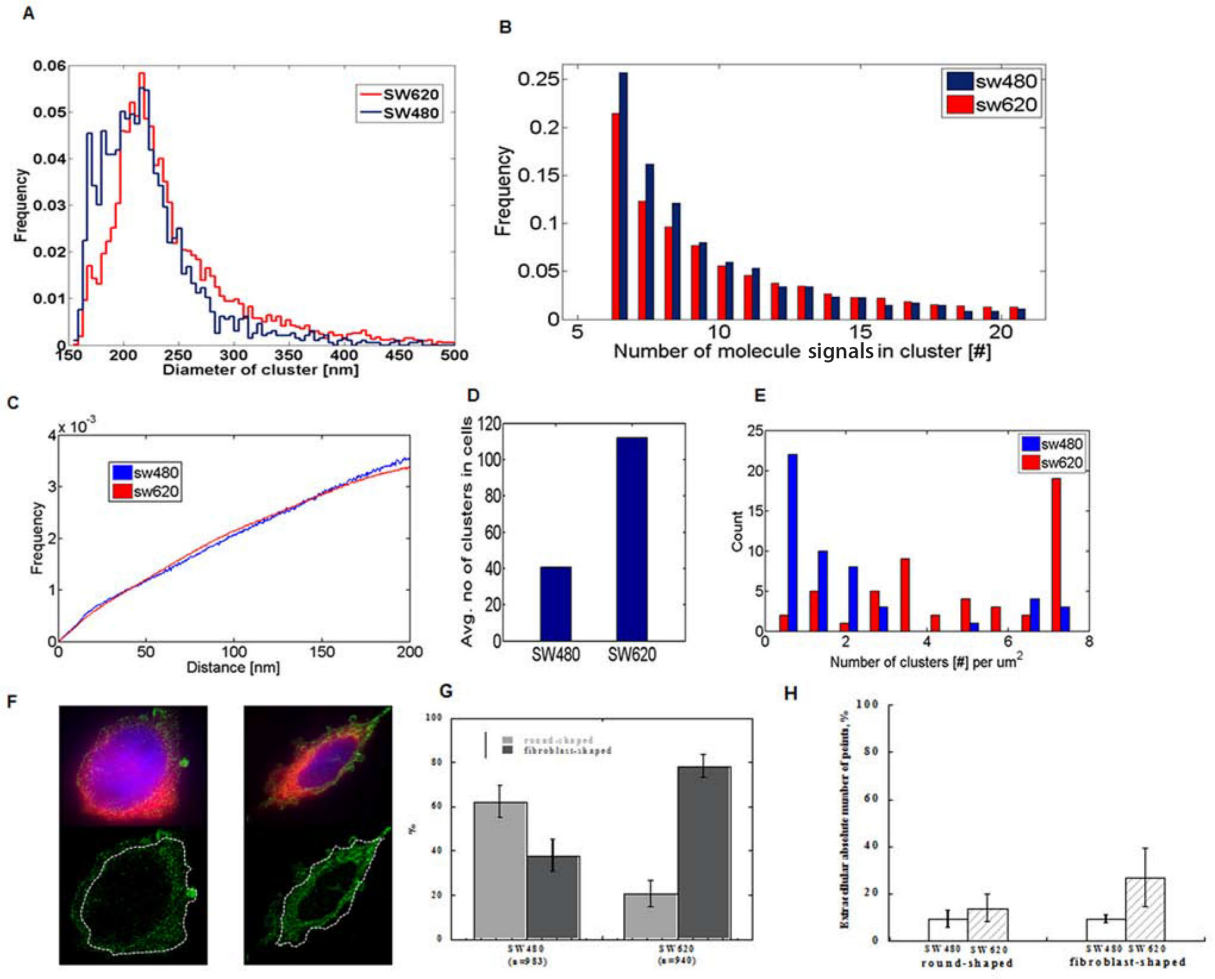
SMLM images of single cells reveal clustering of miR-31 molecules and differential extracellular distribution in low- and highly metastatic CRC cells **A.** Line diagram showing the frequency distribution of observed cluster sizes in SW480 and SW620 cells. Both cell lines are characterized by the same cluster size. **B.** Frequency histogram of miR-31 molecules in individual clusters in the two cell line types showing a wide range in miR-molecule count. **C.** Frequency histogram of densities of miR-31 molecules within clusters in both SW480 and SW620 cells which are almost identical. **D.** Average cluster count in SW480 and SW620 cells showing a significantly higher tally in SW620 cells (40 in SW480 vs 110 in SW620 cells). **E.** Cluster density distribution of miR-31 molecules in SW480 and SW620 cells showing a higher number of clusters/μm^2^ cell surface area in SW620 cells. SW620 cells have a smaller surface area than SW480 cells (see text). **F.** Representative example of the subcellular distribution of miR-31 molecules in a cell; cytoplasm (blue), plasma membrane (red), and the SMLM reconstruction of miR-31 molecules are represented in green. The dashed white lines show plasma membrane boundaries. Scale bar 1 μm. **G.** Both cell lines are characterized by round and fibroblast shapes (SW480 predominantly round, SW620 predominantly fibroblast). SW620 cells were found to have a higher number of extra-cellular miRs in both sub-populations **H.** Relative quantities of extracellular miR-31 molecules in the round and fibroblast shaped sub-populations of SW480 and SW620 cells.

We employed cluster and segmentation analysis to investigate differences in the subcellular distribution of miR-31 molecules in colorectal cancer cells with high and low metastatic potential. To define potential miR-31 clusters, we used home-written software in Matlab, as described by Kaufmann et al. [23]. The cluster analysis was implemented with a critical density of 132 single molecules per μm^2^ in a cluster, which is equivalent to a minimum of five neighbour molecule signals within a radius of 120 nm. Since we already observed a clustering pattern with conventional microscopy, SMLM was used to further zoom into these clustered structures and analyse the substructures within. In comparison with random data, both cell lines showed a distribution of miR-31-molecules that was significantly non-random (Supplementary Figure S4). The size of the individual clusters was marginally different with an average diameter of 220.53 ± 48.52 nm in SW480 and 242.30 ± 60.11 nm in SW620 cells (Figure 3A). Within individual clusters, the number of detected miR-31 single molecule signals was almost the same in both cell lines (Figure 3B), resulting in comparable miR-31 signal densities within the clusters (Figure 3C). The two cell types are characterized by inherent differences in their shapes and sizes, with the SW480 cells having on average a much bigger surface area (19.68 μm^2^) as compared to 13.56 μm^2^ for SW620 cells. Interestingly, we found the average number of clusters in the SW620 cells to be significantly higher, 111 clusters as compared to 40 clusters in the SW480 line (Figure 3D). This resulted in a significantly higher cluster density (clusters/area) in the SW620 cells, since the cells were smaller and had many more clusters (Figure 3E). Similar results were obtained for miR-21 with the exception that both cell lines had lower cluster density than was observed for miR-31(Supplementary Figure S5).

For further segmentation analysis, the plasma membrane was stained with Cell Mask Deep Red and wide-field images of the plasma membrane of single cells were acquired in addition to the SMLM-stack (Figure 3F). The absolute numbers of detected points in the extracellular compartment of every cell was then evaluated with “cellSegm”, an in-house-written program in Matlab. We also took into consideration that both SW480 and SW620 cell lines have two subpopulations of cells with round- and fibroblast- morphologies, respectively (Figure 3G), which are characterized by different mobilities [20]. The SW620 cells had on average more miR-31 molecules in the extracellular compartment than the SW480 cells (Figure 3H).

### Exosome-associated localization of miR-31 in highly metastatic versus low-metastatic human colorectal cancer cells

As was evident from the segmentation analysis, both SW480 and SW620 cell lines had relatively large numbers of detected miR-31 signals in the extracellular space. We interpreted this as supportive evidence for the postulation that miRNAs are released from cells in microvesicles, such as exosomes, or within vesicle-free argonaute-protein complexes [24–28].

**Figure 4:**
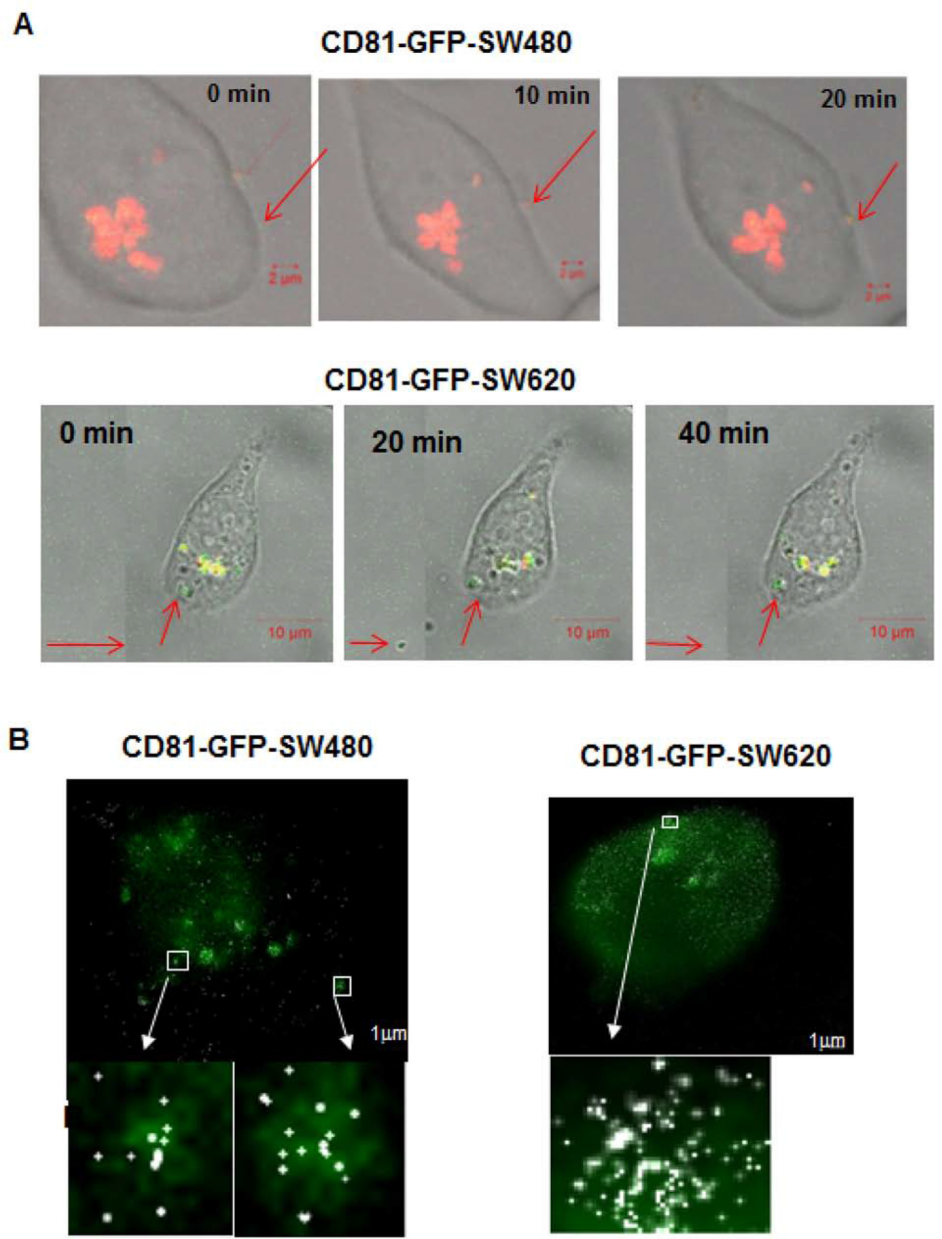
Confocal time-lapse live-cell imaging of exosome traced CD81-GFP-SW480 and CD81-GFP-SW620 cells transfected with an Alexa568-tagged miR-31 probe **A.** Release of vesicle-like structures in CD81-GFP-SW480 cells; scale bar 2 μm. **B.** Budding of vesicle-like structures containing miR-31 molecules in CD81-GFP-SW480 and –SW620 cells during cell division. Scale bar 2 μm. The red arrows show observed events during time-lapse live-cell imaging.

Moreover, since fibroblast shaped cells are generally characterized by higher mobility, an indicator of higher aggression and higher metastatic propensity, we postulated that the more metastatic SW620 cells released more miRs extracellularly. To evaluate the secretion of miR-31 in exosomes, we used the exosome secretion cyto-tracer lenti vector pCT-GFP-CD81 (Biocat GmbH, Germany) in both SW480 and SW620 cells [29]. We observed vesicle-like structures in fixed and live cells by confocal microscopy outside the cells including their active release in both SW480 and SW620 cells (Supplementary Figure S6). Using SMLM, we superimposed exosome-tagged GFP signals acquired with wide-field microscopy with the reconstruction images of miR-31-molecules, enabling us to obtain an overlap of miR-31-signals within exosomes within and outside the cell. The images thus confirmed that labelled miR-31molecules enter exosomes akin to native miRNAs (Figure 4). Additionally, the cellular and exosomal fractions were analysed by Western blotting (data not shown), where both the cellular and exosomal fractions of SW480 and SW620 confirmed the presence of CD81 and GFP.

### Detection and co-localization of miR-31 in isolated exosomes of highly metastatic versus low-metastatic human colorectal cancer cells

**Figure 5:**
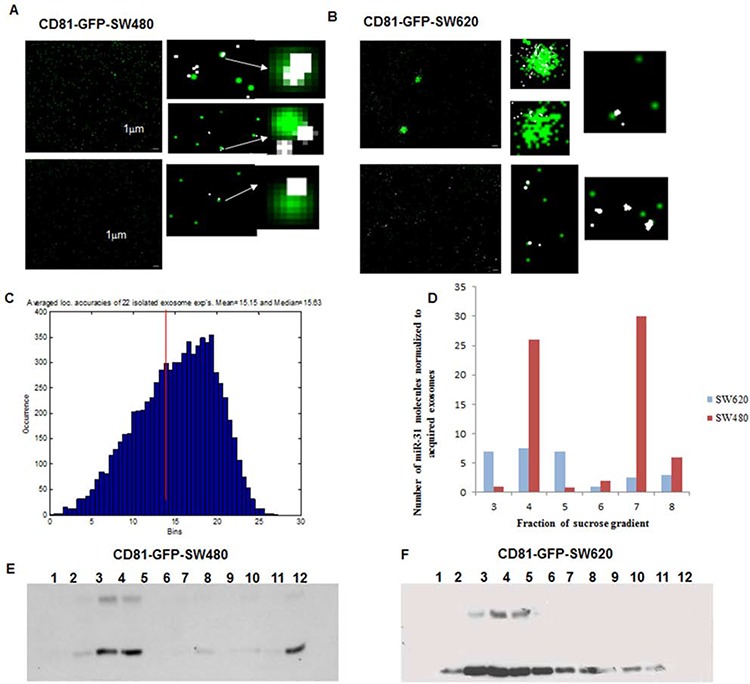
SMLM images of isolated exosomes and their co-localization with miR-31 in CD81-GFP stable -SW480 and -SW620 cells **A.** and **B.** Representative SMLM reconstruction images of isolated exosomes from CD81-GFP-SW480 and CD81-GFP-SW620 cells, respectively. Both cell lines were seeded overnight and transfected with 10 nM of miR-31 Alexa568-probe in FCS-depleted media for 48 h. The exosomes were then isolated by ultracentrifugation and fixed with 4% PFA. Images were acquired by both wide-field microscopy and SMLM; Scale bar 1 μm. **C.** Localization accuracy histogram of miR-31 SMLM acquired molecule signals in exosomes. **D.** Bar chart showing the number of detected miR-31 single molecule signals in the different fractions of the isolated exosomes. The highly metastatic SW620 cell line had significantly more miR-31 molecules in exosomes that were predominantly seen in fractions 4 and 7, whereas in comparison, the SW480 cell line had fewer miR-31 molecules in exosomes seen across fractions 3–5. **E.** and **F.** Western blot analysis of sucrose gradient fractions with an anti-CD81 antibody for the detection of isolated exosomes in the different fractions. Fractions 3 and 4 are consistent in both cell lines. The lower band at 28KDa represents endogenous CD81 and the band at 45KDa is CD81-GFP.

We considered the possibility that the exosomes we previously isolated using simple ultracentrifugation contained other microvesicles, therefore, we additionally performed ultracentrifugation in a sucrose gradient, which allowed us to separate microvesicles with different densities. Each obtained fraction of GPF-CD81-SW480 and GFP-CD81-SW620 cells was visualized microscopically (Figures 5A and 5B) with comparable localization accuracies to that observed for single cell imaging (Figure 5C). SMLM analysis of the detected miR-31 single molecule signals showed variations in the quantity of miRNA molecules within the respective fractions in both cell lines (Figure 5D). Furthermore, we also analyzed the separate fractions by Western blotting where, as exosomal markers, endogenous CD81 (lower band) and GFP-CD81 (upper band) were detected (Figures 5E and 5F). GFP-CD81 appeared only in fractions 3–5 of both cell lines, with increasing levels of GFP-CD81 in each fraction.

We compared the absolute numbers of detected single miR-31 signals normalized to the amount of acquired GFP-exosomes, in both cell lines with the data of Western blotting analysis and observed a consistent overlap of data. The most abundant fraction of miR-31 in GFP-CD81-SW480-exosomes was found in fraction 4 and that for GFP-CD81-SW620 cells was in fractions 3–5. These data show that whereas both SW480 and SW620 cells generate exosomal microRNAs, the SW480 cells produce exosomes with a higher variation per fraction of miR-31 molecules. The more metastatic SW620 cells, however, on average showed a higher number of miR-31 molecules across the majority of exosomal fractions.

## DISCUSSION

A number of attempts have been made at localization microscopy in the analysis of single molecules in cells [30–33]. These studies made it clear that certain biological questions could only be addressed with particular microscopy techniques, especially in the context of small non-coding RNAs, which, to our knowledge, have been never studied before by localization microscopy.

In this study, we demonstrated a new single-molecule microscopy approach, specifically SMLM, to detect and study small non-coding RNAs. This was accompanied by sequential quantitative analysis of their subcellular distribution and clustering. Using this method, we show that super-resolution localization microscopy can be a very useful tool in investigating the functionality of miRs, and equally demonstrate that miRs are differentially localized and distributed within exosomes at the single molecule level in human cancer cells with different metastatic abilities. SMLM was used to detect single molecules in fixed cells with an organic dye-labelled oligonucleotide probe complementary to miR-31 (and miR-21), in which miR-31 was evaluated as a prototype miRNA molecule important in metastasis.

We are optimistic that our method can contribute significantly to a more complete understanding of the mechanistic properties of particular miRs, especially in understanding their role in cellular communication, but more importantly in dissecting ambiguities and contradictions related to miR function. In the case of miR-31, for example, reports on its validation in metastasis have in part been contradictory with some studies showing an association of high miR-31 expression with poor prognosis in some cancer types [3, 34] but with a favourable prognosis in others [5, 35]. In one study, a low miR-31 expression was found to be essential for the transformation of normal into cancer-associated fibroblasts [36], capable of significantly enhancing progression of cancer cells towards an invasive phenotype, emphasizing that dissecting miR cross talk between different cell types at the subcellular level might be crucial in pining down their exact function in processes such as metastasis.

It is evident that a lot of different factors affect miRNA-mRNA interactions, and we are still far from understanding all of the underlying molecular mechanisms, especially in tumor cells, where aberrant expression of miRs, mRNAs, but also proteins might be involved in target interaction and consequent gene regulation. Molecular tools, such as SMLM that simplify the appreciation of these complex interactions are therefore urgently needed. The quantitative analysis of our SMLM images shows that miR molecules form clusters and we can extrapolate that such clustering is indicative of an additional level in the complexity of gene deregulation. Moreover, since we observed that cluster density is different in cells with different metastatic propensities, we think that the differential clustering of miR-31 single molecules in SW480 and SW620 is biologically relevant, and could be closely related, e.g., to a differential multi- target miRNA interaction with several different mRNAs. Additionally, the outcome of such differential interactions in low versus highly metastatic cancer cells might be enhanced by a combination of both clustering and increased expression. This paper presents the first visualization of a miR in exosomes at the single molecule level, and for the first time shows that highly metastatic colon cancer cells have a significantly higher number of miR-31 molecules in the majority of their exosomal fractions, as compared to poorly metastatic cells of the same genetic background. This hypothetically could indicate an increased propensity, or ability, of highly metastatic cells to accumulate particular miRs in exosomes, which when secreted, would support the metastatic cell to more efficiently communicate with the surrounding stromal cells, and to prime the microenvironment as a metastatic niche. Also, this would certainly enable the metastatic cell, rather than the non-metastatic cell, to efficiently perform long distance communication with distant cells, compartments or organs via accumulated miRNAs like miR-31 in their secreted exosomes.

Taken together, our novel SMLM-approach is the first to significantly detect miR-31 at the single-cell, single-molecule level and could therefore, in contrast to conventional microscopy, be exploited to not only acquire detailed information about the subcellular localization of miR-31 or indeed any other (small) non-coding RNA in individual human cells, but to also understand their interactions in cell-cell communication at a detailed single-molecule level. Also, this method could certainly be of significance in detecting the metastasis-critical or active cell population within a heterogeneous pool of primary cancer cells. Finally, the SMLM technique that we have used in the present work could easily be expanded to other biological applications that would allow us to study biological nanostructures and their critical interactions in depth in highly critical processes not limited to cancer metastasis.

## MATERIALS AND METHODS

### Cell culture

The SW480 human primary colorectal cancer cell line and the SW620 lymph node metastasis cell line were cultured in RPMI and L-15 media (Invitrogen Corporation, Carlsbad, CA, USA), respectively, supplemented with 10% fetal calf serum (FCS, PAA Laboratories GmbH), 100 U/mL penicillin and 0.1 mg/mL streptomycin. All cells were grown at 37°C in 5% CO2 and 90% humidity. All cell lines including those whose RNA was used for RT-PCR were obtained from the American Type Culture Collection (ATCC) and were re-authenticated by the German Collection for Microorganisms and cell culture (DSMZ), Germany.

### Transfection and staining

For SMLM, cells were seeded at a density of 2*10^5^ cells/well, in 6-well plates on coverslips (24 × 24 mm, #1.5, Menzel-Gläser, Braunschweig, Germany) and allowed to grow and attach overnight. The cells were transfected using Metafectene (Biontex Laboratories GmbH, Martinsried, Germany) with an SMLM suitable 5′-end photoswitchable Alexa568 fluorophore labelled RNA oligonucleotide probe, with a sequence complementary to the human mature miR-31 (IBA Gmbh, Göttingen, Germany). 24 hours after transfection, the cells were washed with PBS, and for segmentation analysis, stained with 1.5 μg/ml CellMask DeepRed (Life Technologies, Thermo Fisher Scientific, Waltham, USA) for 3 min, rinsed with PBS and finally fixed with 4% paraformaldehyde in PBS for 10 min. The cells were subsequently washed 5 times in PBS for 3 min each and then stained with DAPI (Invitrogen, Thermo Fisher Scientific, Waltham, USA) according to manufacturer's instructions. Fluoromount-G (SouthernBiotech, Birmingham, USA) was used as an embedding medium.

Isolated exosomes were plated on freshly prepared poly-L-lysine coated coverslips and mounted on slides by dropping the embedding medium Fluoromount-G.

### RNA isolation, cDNA preparation and Real-time PCR

Total RNA (including small RNAs) was isolated from the following cancer cell lines; SW480, SW620, SW48, Colo320, WiDr, DLD1, HCT-15, HT-29, Caco-2, RKO, HCT-116 and Lovo and GEO using the miRNeasy kit (Qiagen, Germany) according to the manufacturer's guidelines. Complementary DNA for RT-PCR was synthesized using the miScript II RT kit (Qiagen, Germany) with 500 ng of total RNA.

Quantitative real-time PCR (qRT-PCR) was done on the LightCycler480 (Roche), using the SYBR green detection system (Thermo Scientific). The miR-31 and miR-21 primers were purchased from Qiagen from the Quantitect Primer Assay collection (Qiagen, Germany). Relative expression was calculated using the δδCT method, with normalization to RNU6 or/and SNO72.

### Exosome purification and sucrose density gradient fractionation

The indicated cell lines were cultivated in the appropriate FCS-depleted media for 48–72 hrs. The cell supernatants were collected and exosomes were pelleted by ultracetrifugation at 110,000 g for 2 h at 4°C [37]. Sucrose-density-fractionation was performed as previously described [38]. Basically, 100 μg vesicles in 100 μl PBS were loaded on top of a stepwise sucrose gradient. The layers had the following concentrations: 2 M, 1.3 M, 1.16 M, 0.8 M, 0.5 M and 0.25 M in TBS. The gradient was centrifuged for 2.5 h at 100,000 g. Twelve 1 ml fractions were collected from the top of the gradient and used for subsequent analysis.

### Lentiviral infection

SW480 and SW620 cells were infected with the exosome secretion cyto-tracer lentiviral vector, pCT-GFP-CD81 (Biocat GmbH, Germany) using the TransDux^TM^ protocol (System Biosciences, Mountain View CA). Positive cell clones were selected by flow cytometry and propagated in culture using puromycin as a selection marker.

### Conventional microscopy

Conventional, including confocal microscopy of fixed cells and live-cell imaging (in phenol red-free Opti-MEM media, Life Technologies, USA) was done with the Zeiss LSM 700, LSM 710 ConfoCor 3 and the Zeiss Cell Observer Microscope, as appropriate (Carl Zeiss Microscopy GmbH, Jena, Germany).

### Single-Molecule Localization Microscopy (SMLM) setup

Localization microscopy was performed on a custom built microscope, in which the original iMIC microscopy core (FEI Munich GmbH, Germany) was upgraded. The optical system was equipped with 3 different objectives: 20X, 40X low magnification objectives, and a 100X with 1.46 NA oil immersion objectives (Zeiss alpha Plan-Apochromat, Carl Zeiss Microscopy GmbH, Jena, Germany). We routinely used low magnification objectives to scan the slide and proceeded with the 100X objective in selecting single cells. Specimens were laterally positioned on a motorized stage (Applied Scientific Instrumentation, Eugene, Oregon, USA). The microscope was equipped with a custom made thermo-stability water-based control to provide thermal and mechanical stability to the optical system.

The optical system is equipped with four lasers with wavelengths 405 nm (120 mW), 491 nm (200 mW), 561 nm (200 mW) and 642 nm (140 mW), which are combined in one line using LightHUB-4 (Omicron-Laserage Laserprodukte GmbH, Rodgau-Dudenhofen, Germany).

A light beam (green line, Figure 2A) passing through an acousto-optical tunable filter (AOTFnC-400.650 AO tunable Filter, AA OptoElectronic, Orsay, Cedex, France) is reflected by a broadband dielectric mirror (BB1-E02, Thorlabs Inc, Newton, New Jersey, USA), and then the combination of a beam expander and beam shaping system creates a homogenous illumination using different optomechanical components (including optomechanics components from Standa Ltd, Vilnius, Lithuania). Thereafter, the homogenized laser beam is directed to the focusing lens that is deflected by a dichroic beam splitter (Quad Filter Set: Reflection 375–405/488/561/640, AHF, Tübingen, Germany) finally reaching the high numerical objective with high density (1–10 kW/cm^2^) laser power in the object plane.

The emission light (red line, Figure 2A) from the specimen is received by the objective and then separated from the excitation light by a dichromatic beam splitter and by an additional blocking filter (Quad Filter Set: Transmission 446/523/600/677, AHF, Tübingen, Germany) shifting to a 1X tube lens (Carl Zeiss Microscopy GmbH, Jena, Germany).

Subsequently, the fluorescent light is directed by a mirror inside the iMIC microscopy core to the custom modified beam splitting optics with 2X magnification (Andor TuCam, Andor Technology Ltd., Belfast BT12 7AL, UK) that satisfy the Nyquist theorem for optical resolution. The beam is captured by one of the two electron multiplying charge-coupled device cameras (Andor iXon Ultra 897, Andor Technology Ltd., Belfast, UK), which are adjusted using a precision mechanics table (LT 60–50, OWIS GmbH, Staufen/Germany). The software hub of the entire system including data acquisition is based on the Live Acquisition Software (FEI Munich GmbH, Germany). The resolution of the SMLM system was validated using the commercially available nanoruler (Steinhauer et al., 2009), which contains specially folded DNA molecules labelled with Alexa Fluor 647, with the fluorophores arranged in pairs at a distance of 35 ± 5 nm (NanoRuler LM35-Alexa647, STS Nanotechnology UG, Germany).

### SMLM imaging and reconstructions

Wide-field images were acquired with an EMCCD camera at minimum laser power density to avoid bleaching. DAPI, GFP, Alexa568, CellMask DeepRed were excited at 405 nm, 491 nm, 561 nm and 642 nm, respectively. Finally, an SMLM-stack of 3000 frames with an EM gain between 100 and 300 with 50ms integration time was taken. The fluorescent signal of light-induced fluorophores was acquired using the Live Acquisition Software (FEI Munich GmbH, Germany) which records 3000 image frames during a time lapse.

The SMLM-stack was reconstructed with a custom written software in Matlab (Matlab, The Mathworks, Natick, Massachusetts, USA) [19] based on a 2D Gaussian fit using the Levenberg-Marquardt algorithm. At first, the tally numbers were converted to photon numbers and then the differential stack was calculated by subtracting a succeeding frame from a preceding one. The data points were then fitted to a 2D Gaussian distribution to determine the positions of the single detected molecules and generate an SMLM-reconstruction image by Gaussian blurring of each position, corresponding to the individual localization accuracy.

### Cluster analysis

Cluster analysis was implemented using home-written software based on an algorithm established by Kaufmann and colleagues [18] using the Matlab DipImage package for image processing. The critical density for a cluster was set to 530 points per μm^2^, with a defined minimum of five neighbor molecule signals surrounding each detected single molecule within a radius 60 nm.

### Segmentation analysis

The “cellSegm” program was designed to allow the user to analyze the distribution of molecules of interest using SMLM acquired signals. Based on a wide-field image of the cell in which the membrane is delineated with a stain analyzed along with the SMLM stack, the user selects three areas by eye corresponding to the nucleus, cell membrane and cytoplasm, and the extracellular region. This ensures that the selection of the regions is unbiased irrespective of the distribution of the molecules of interest. The user first enters the wide-field images and reconstructs localization images and the Orte (position) precision matrices of the data sets to be analyzed. We applied segmentation analysis to discriminate the plasma membrane by fluorescent staining, and then measured the number of single molecule signals in the extracellular region of the cells. In our case, this was done via three input fields. For the scaling, a bilinear interpolation algorithm was implemented. After segmenting the regions, the results of the analysis were printed on a screen.

### Reporter gene assay

The cMET 3′UTR plasmid was a kind gift from Dr. Stefan Eichmüller (German Cancer Research Center), and contained the cMET 3′UTR in a psi-CHECK2 vector backbone cloned downstream of an SV40 promoter-driven Renilla luciferase gene. Cells were plated at a density of 3 × 10^4^ cells/well in 96-well plates (Nunc), and were transfected after a 24 h seeding period using Metafectane (Biontex Laboratories). 10–50 nM of labelled miR-31 probe or mimic control plasmids were co-transfected with 50 ng of the psi-CHECK-2 MET 3′UTR luciferase reporter plasmid. 24 hrs after transfection, the cells were washed with DPBS and lysed with 20 μl of 1X passive lysis buffer (Promega Corporation, USA) on a rotary shaker for 30 minutes at RT. Assays were conducted in quadruplicates and repeated at least 3 times. Reporter signals were assessed with the Dual-Luciferase Reporter Assay system (Promega, USA)). 50 μl of the Luciferase Assay Reagent II solution was added directly to each well, followed by measurements of firefly luciferase, and then addition of Stop and Glo reagent and measurement of renilla luciferase activity on a microplate reader (TECAN Trading AG, Switzerland).

### Flow cytometry analysis and sorting

Cells were analysed with a FACSCalibur flow cytometer and CellQuest software (BD Biosciences). Viable cells were identified by propidium iodide exclusion. GFP-positive cells were sorted on a FACSAria flow cytometer (BD Biosciences).

### Western blotting

Protein lysates were obtained by centrifugation and solubilization in RIPA buffer (50 mM Tris–HCl pH 7.5, 150 mM NaCl, 0.1% SDS, 1% sodium deoxycholate (DOC), 1% Triton X-100, 1:50 protease inhibitors cocktail (Complete, Roche)), for 10 min. Protein concentrations were determined with the BCA protein assay kit (Thermo Scientific), and samples were separated by SDS-PAGE.

### Statistical calculations

Basic calculations and plots were made using GraphPad Prism (5.0), Microsoft Excel and KaleidaGraph (Version4.1), as appropriate.

## SUPPLEMENTARY FIGURES


