# Supplementary material for: Single-Molecule Localization Microscopy allows for the analysis of cancer metastasis-specific miRNA distribution on the nanoscale
Source: Oncotarget. 2015 Nov 5;6(42):44745–57. doi: 10.18632/oncotarget.6297 (PMC4792589; doi:10.18632/oncotarget.6297)
Supplement: Supplementary file 1 [file oncotarget-06-44745-s001.pdf]

SUPPLEMENTARY FIGURES

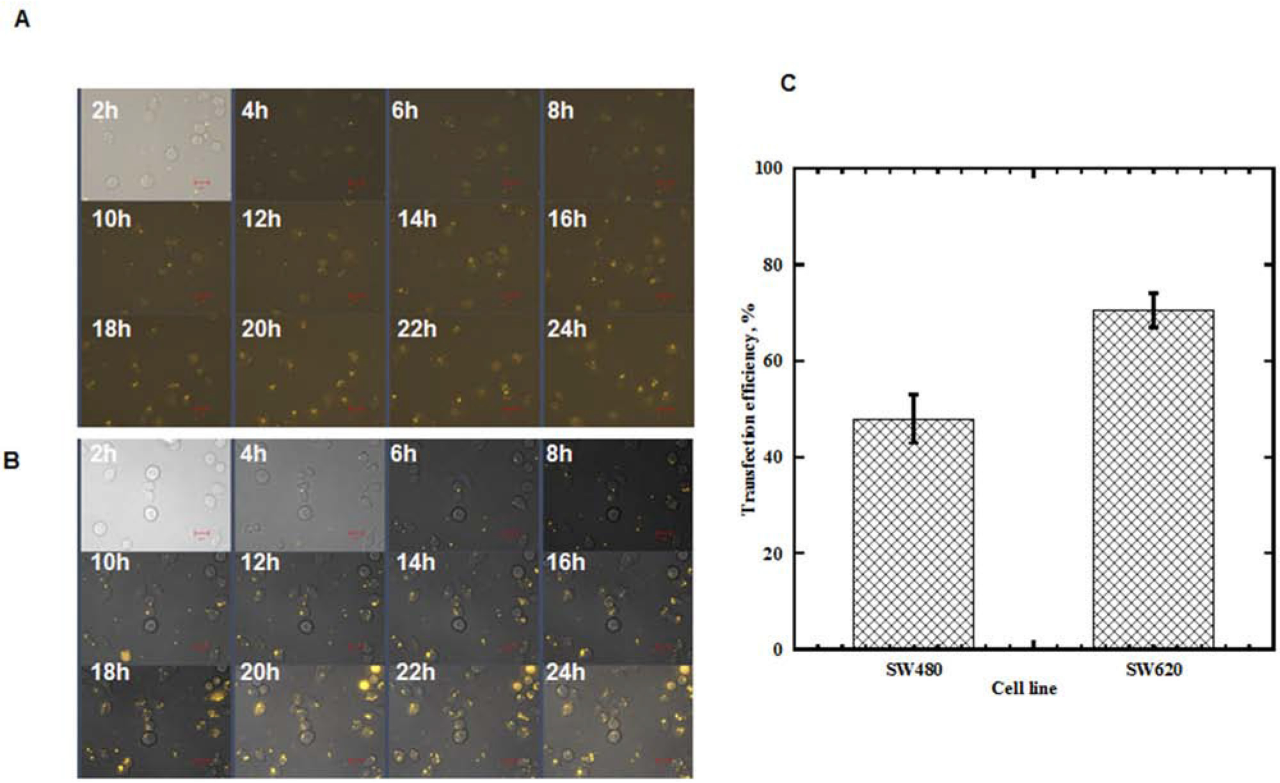

Supplementary Figure S1: Time lapse evaluation of transfection efficiency in SW480 and SW620 cell lines. A, B. SW480 and SW620 cells were transfected by Alexa 568 labelled miR-31 oligonucleotide probe and the signals were monitored over 24 hrs in each cell line. The transfection efficiency in SW620 cells (B) was slightly, but not significantly higher than that in SW480 cells (A), **C.** is a graphical representation of (A) and (B).

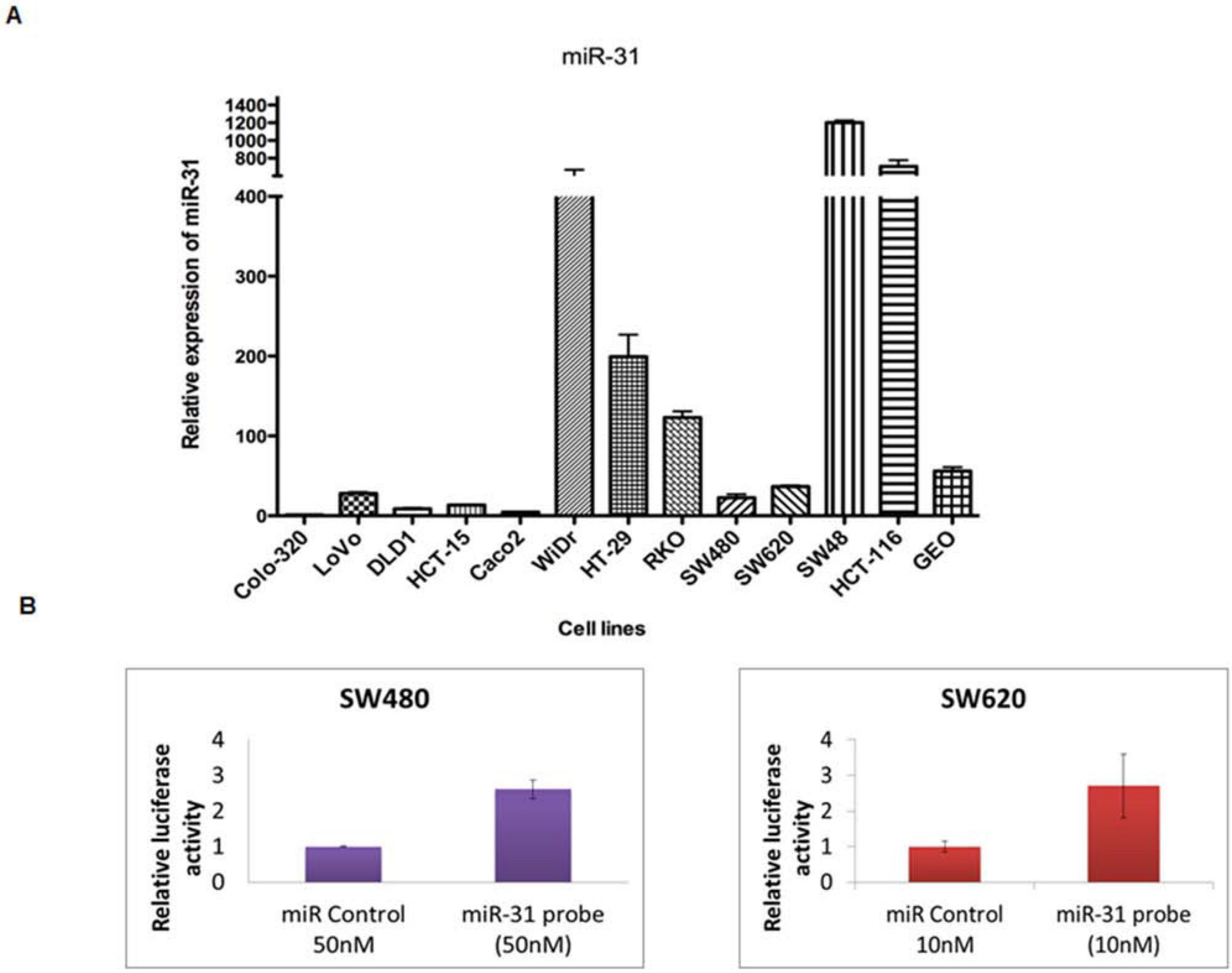

Supplementary Figure S2: Analysis of endogenous miR-31 expression and confirmation of Alexa568 tagged miR-31-probe specificity. A. Endogenous expression of miR-31 in a panel of colorectal cancer cell lines. Expression values are shown relative to the Colo320 cell line, which had the highest ct values (lowest expression) in the panel tested. SW620 cells have a higher expression of miR-31 than SW480 cells. All expression values were normalized to the RNU6 house-keeping small RNA. **B.** Luciferase reporter assays for SW480 and SW620 cells transfected with the psi-CHECK-2 cMET 3′UTR and a scrambled control or miR-31-Alexa568-probe. The renilla luciferase activity was normalized to the internal control firefly luciferase activity within each sample. The assay was conducted in quadruplicate in three independent experiments.

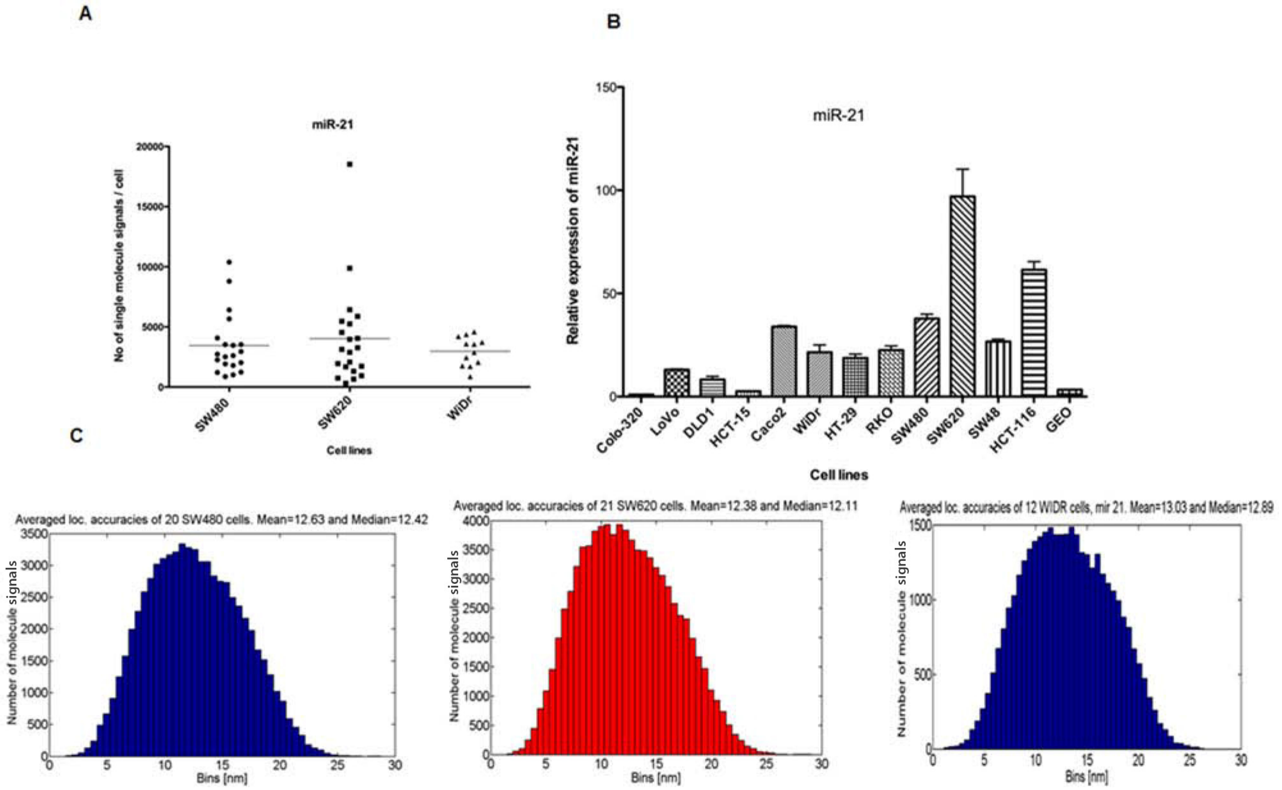

Supplementary Figure S3: Alexa568 labelled miR-21 control experiments. A. Dot plot representation of reconstructed miR-21 single molecule signals as obtained with SMLM in SW480, SW620 and WiDr cells transfected with Alexa 568 labelled miR-21. The cells were transfected with 10 nM of probe and images were acquired after 24 hrs of transfection (as for miR-31). Mean signal intensity values/cell show that SW620 have the highest expression of miR-21, followed by SW480, and WiDr. These results are corroborated in **B.** which shows the endogenous expression of miR-21 in the same panel of colorectal cancer cell lines tested for miR-31. Expression values are also relative to Colo320. **C.** The localization accuracies for miR-21 were calculated in SW480, SW620 and WiDr cell lines and show comparable results in all three cell lines. These results also closely reflect the localization accuracy of miR-31 in SW480 and SW620 cells (Figure 2 of manuscript).

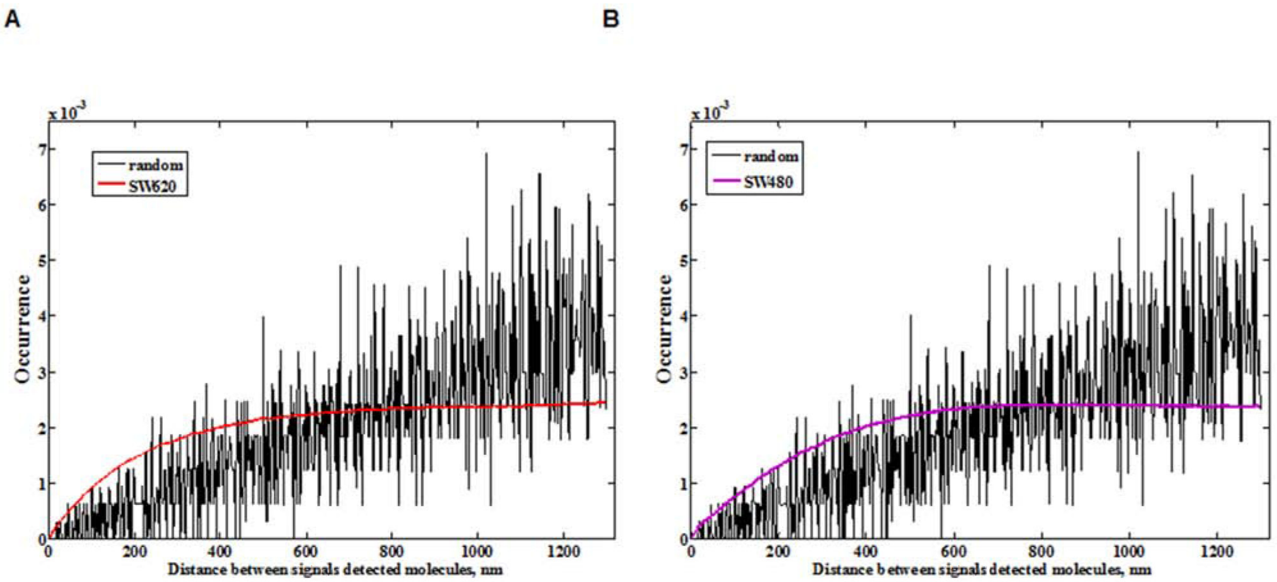

Supplementary Figure S4: Comparison of the subcellular distribution miR-31 detected single molecules within clusters with random distribution. A, B. All distances up to 1300 nm between the individual molecules occurring in the SMLM-measurements SW480 (purple) and SW620 (red) were analyzed. The distribution pattern of the detected miR-31 molecules in both SW480 and SW620 cells were different from random distribution (black lines). The graphs show data from 5 independent experiments.

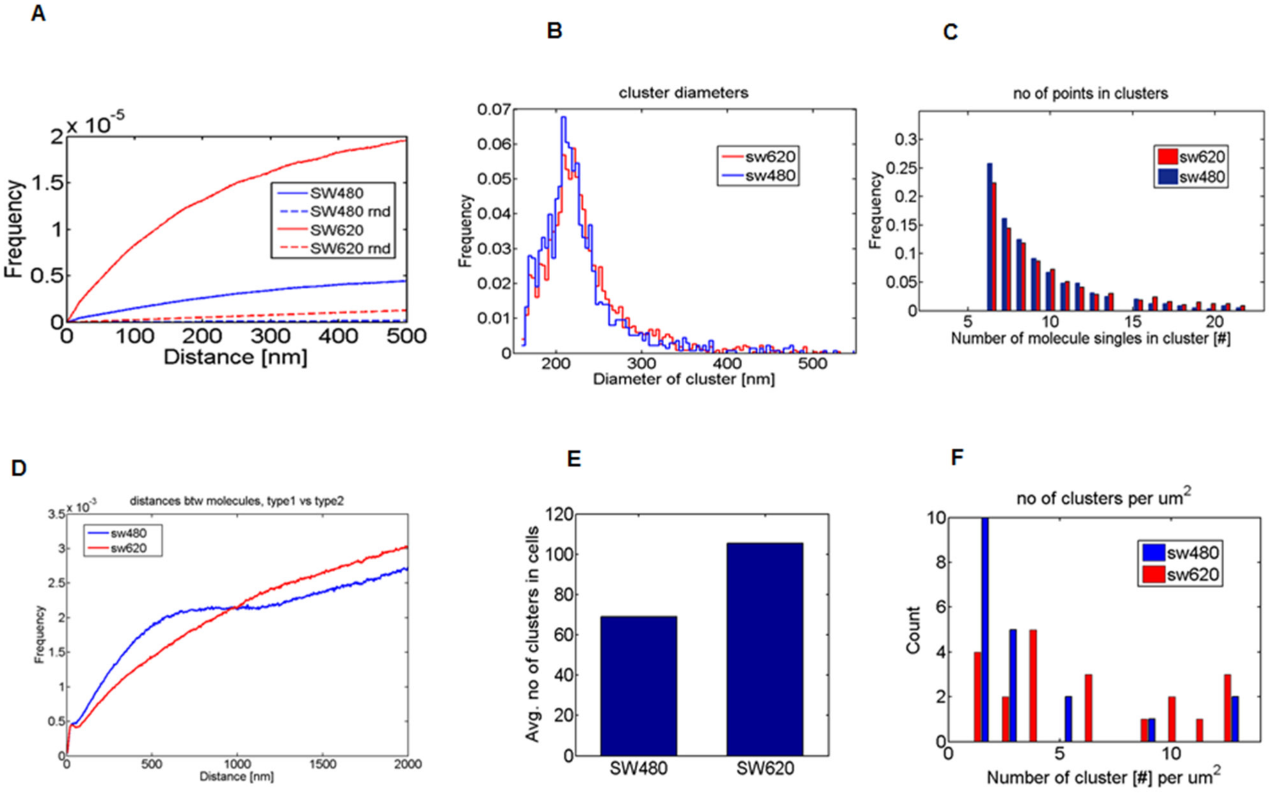

Supplementary Figure S5: Cluster analysis of miR-21 in SW480 and SW620 colorectal cancer cell lines. A. Line diagram showing the distribution curve of miR-21 molecules in SW480 and SW620 cells. All distances up to 500 nm between the individual molecules occurring in the SMLM-measurements SW480 (continuous blue line) and SW620 (continuous red line) were analyzed. The distribution pattern of the detected miR-21 molecules in both SW480 and SW620 cells were different from random distribution (dashed blue and red lines). **B.** Line diagram showing the frequency distribution of observed cluster sizes in SW480 and SW620 cells. Both cell lines are characterized by the same cluster size. **C.** Frequency histogram of miR-21 molecules in individual clusters in the two cell line types showing a wide range, but similar number of miR-molecules in clusters. **D.** Frequency histogram of densities of miR-21 molecules within clusters in both SW480 and SW620 cells showing SW480 with a higher number of compact clusters and SW620 with a higher number of sparse clusters. **E.** Average cluster count in SW480 and SW620 cells showing a significantly higher number in SW620 cells. **F.** Cluster density distribution of miR-21 molecules in SW480 and SW620 cells showing a higher number of clusters/μm^2 cell surface area in SW480 cells.

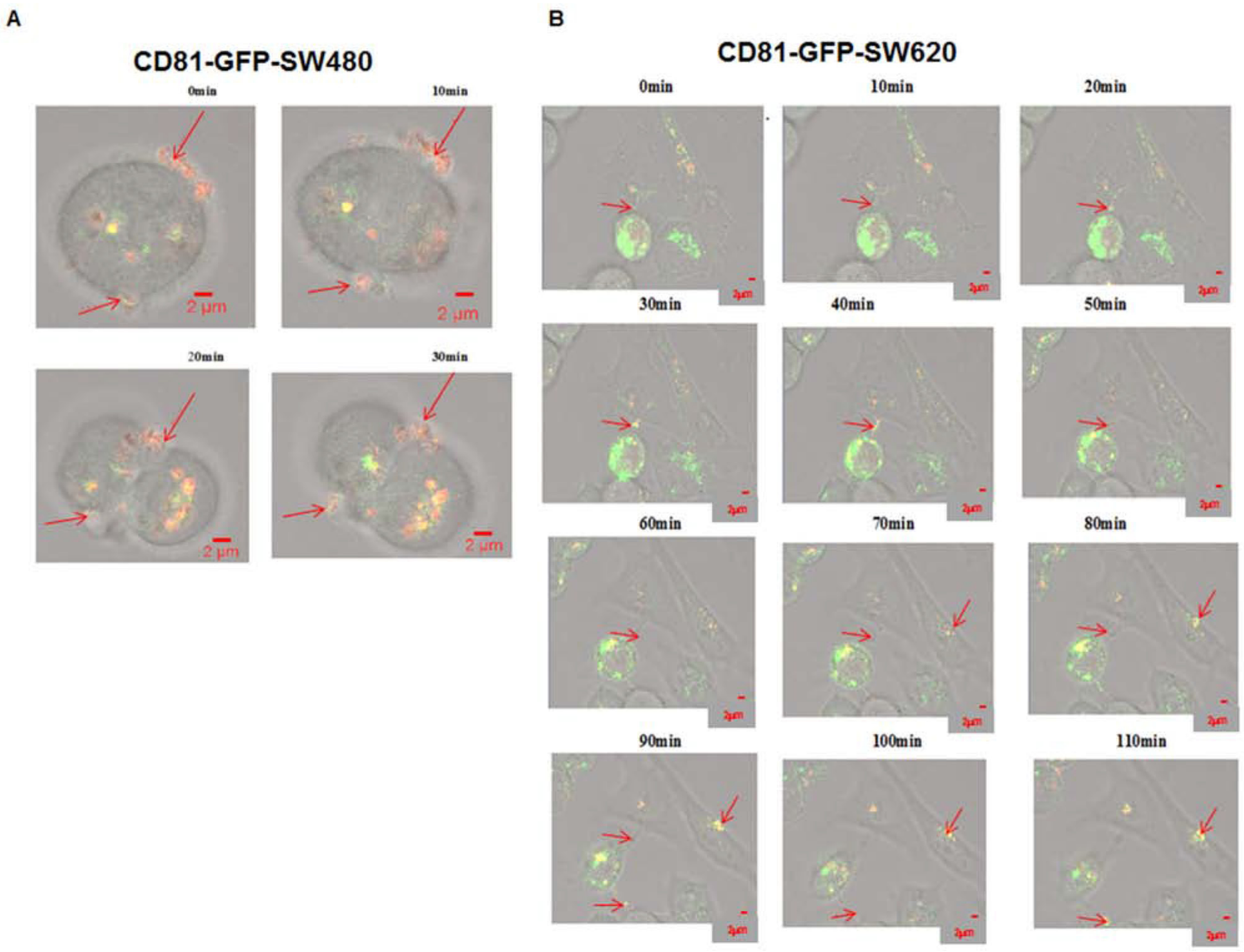

Supplementary Figure S6: Exosome-associated miR-31 localization in low-metastatic CD81-GFP-SW480 and metastatic CD81-GFP-SW620 cells. A. and **B.** Confocal time-lapse live-cell imaging of Alexa568- tagged (red) miR-31 transfected SW480 and SW620 cells. The cells stably expressed a CD81 GFP vector for the identification of exosomes; scale bar 2 μm (CD81-GFP-SW480) and 10 μm (CD81-GFP-SW620). Releases of vesicles-like structures are indicated with red arrows.
